# Supplementary material for: Increased Phosphatase of Regenerating Liver-1 by Placental Stem Cells Promotes Hepatic Regeneration in a Bile-Duct-Ligated Rat Model
Source: Cells. 2021 Sep 24;10(10):2530. doi: 10.3390/cells10102530 (PMC8533985; doi:10.3390/cells10102530)
Supplement: Supplementary file 1 [file cells-10-02530-s001.zip › cells-1359822-SI.pdf]

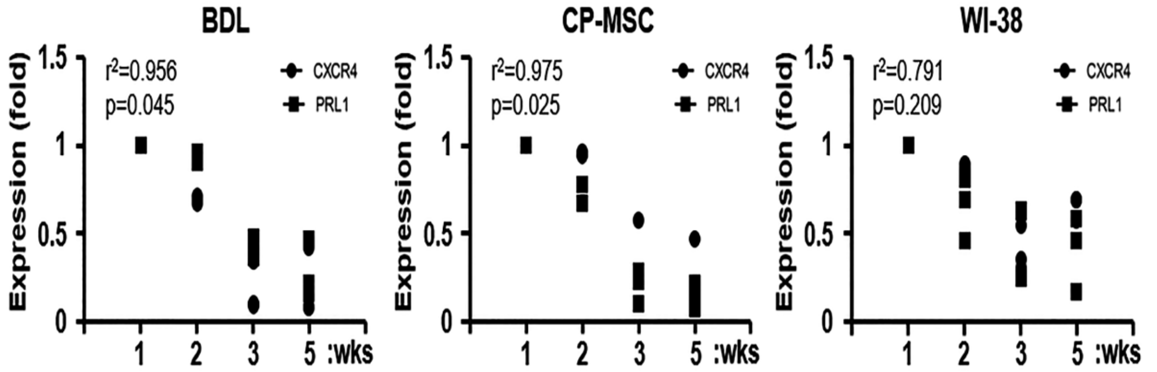

Figure S1: Correlation analysis between CXCR4 and PRL-1 expression in liver tissue of the BDL, CP-MSC, and WI-38 groups.
